# Supplementary material for: Associations of Neutrophil-to-Lymphocyte Ratio, Platelet-to-Lymphocyte Ratio and Monocyte-to-Lymphocyte Ratio with Osteoporosis and Incident Vertebral Fracture in Postmenopausal Women with Rheumatoid Arthritis: A Single-Center Retrospective Cohort Study
Source: Medicina (Kaunas). 2022 Jun 26;58(7):852. doi: 10.3390/medicina58070852 (PMC9321011; doi:10.3390/medicina58070852)
Supplement: Supplementary file 1 [file medicina-58-00852-s001.zip › medicina-1761450-Supplementary.pdf]

Supplementary

Table S1. Association of baseline platelet-to-lymphocyte ratio and monocyte-to-lymphocyte ratio with osteoporosis in patients with rheumatoid arthritis.

Dependent Variable	Hip OP		Hip OP	
Independent Variable	High Baseline PLR		High Baseline MLR	
	OR (95% CI)	*p*-Value	OR (95% CI)	*p*-Value
Model 1	2.54 (1.44–4.49)	0.001	1.8 (1.05–3.1)	0.034
Model 2	2.42 (1.3–4.52)	0.005	1.6 (0.88–2.92)	0.123
Model 3	2.31 (1.23–4.33)	0.009	1.57 (0.86–2.89)	0.14
Model 4	2.3 (1.21–4.36)	0.011	1.65 (0.89–3.06)	0.111

Model 1: no variable adjusted. Model 2: age and BMI adjusted. Model 3: age, BMI, DAS28-ESR, cumulative GCs dose and calcium/vitamin D adjusted. Model 4: age, BMI, DAS28-ESR, cumulative GCs, calcium/vitamin D, RF positivity, disease duration and DMARDs adjusted. PLR platelet-to-lymphocyte ratio, MLR monocyte-to-lymphocyte ratio, OR odds ratio, CI confidence interval, BMI body mass index, DAS28-ESR disease activity score assessed using the 28-joint count for swelling and tenderness-erythrocyte sedimentation rate, GCs glucocorticoids, RF rheumatoid factor, DMARDs disease modifying anti-rheumatic drugs.

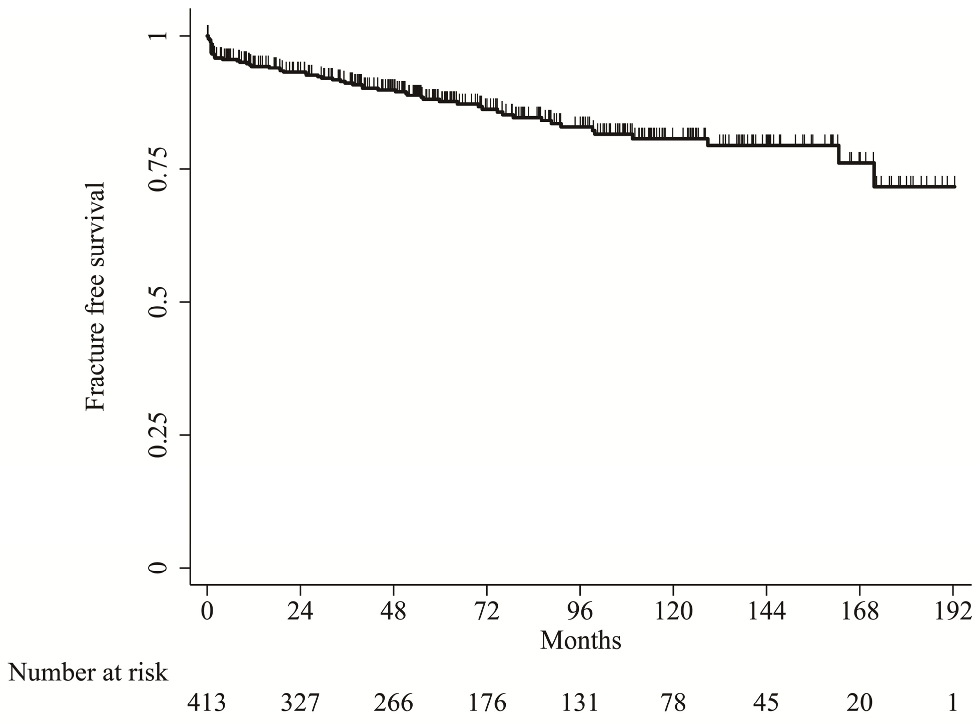

Figure S1. Fracture-free survival of patients with RA.
